# The cytosolic DNA sensor AIM2 promotes *Helicobacter*‐induced gastric pathology *via* the inflammasome

**DOI:** 10.1111/imcb.12641

**Published:** 2023-04-19

**Authors:** Ruby E Dawson, Virginie Deswaerte, Alison C West, Ekimei Sun, Georgie Wray‐McCann, Thaleia Livis, Beena Kumar, Emiliana Rodriguez, Cem Gabay, Richard L Ferrero, Brendan J Jenkins

**Affiliations:** ^1^ Centre for Innate Immunity and Infectious Diseases Hudson Institute of Medical Research Clayton VIC Australia; ^2^ Department of Molecular and Translational Science, Faculty of Medicine, Nursing and Health Sciences Monash University Clayton VIC Australia; ^3^ Department of Anatomical Pathology Monash Health Clayton VIC Australia; ^4^ Pathology and Immunology Department CMU/University of Geneva Geneva Switzerland; ^5^ Department of Microbiology, Biomedicine Discovery Institute Monash University Clayton VIC Australia

**Keywords:** Absent in melanoma 2 (AIM2), gastritis, *Helicobacter*, inflammasome

## Abstract

*Helicobacter pylori* (*H. pylori*) infection can trigger chronic gastric inflammation perpetuated by overactivation of the innate immune system, leading to a cascade of precancerous lesions culminating in gastric cancer. However, key regulators of innate immunity that promote *H. pylori*–induced gastric pathology remain ill‐defined. The innate immune cytosolic DNA sensor absent in melanoma 2 (AIM2) contributes to the pathogenesis of numerous autoimmune and chronic inflammatory diseases, as well as cancers including gastric cancer. We therefore investigated whether AIM2 contributed to the pathogenesis of *Helicobacter*‐induced gastric disease. Here, we reveal that AIM2 messenger RNA and protein expression levels are elevated in *H. pylori*–positive *versus H. pylori*–negative human gastric biopsies. Similarly, chronic *Helicobacter felis* infection in wild‐type mice augmented *Aim2* gene expression levels compared with uninfected controls. Notably, gastric inflammation and hyperplasia were less severe in *H. felis*–infected *Aim2* ^*−/−*^ *versus* wild‐type mice, evidenced by reductions in gastric immune cell infiltrates, mucosal thickness and proinflammatory cytokine and chemokine release. In addition, *H. felis*–driven proliferation and apoptosis in both gastric epithelial and immune cells were largely attenuated in *Aim2* ^*−/−*^ stomachs. These observations in *Aim2* ^*−/−*^ mouse stomachs correlated with decreased levels of inflammasome activity (caspase‐1 cleavage) and the mature inflammasome effector cytokine, interleukin‐1β. Taken together, this work uncovers a pathogenic role for the AIM2 inflammasome in *Helicobacter*‐induced gastric disease, and furthers our understanding of the host immune response to a common pathogen and the complex and varying roles of AIM2 at different stages of cancerous and precancerous gastric disease.

## Graphical abstract

*Helicobacter pylori* infection can trigger chronic gastric inflammation perpetuated by overactivation of the innate immune system which leads to a cascade of precancerous lesions, culminating in gastric cancer. Here, we report that the innate immune DNA sensor absent in melanoma 2 (AIM2) promotes *Helicobacter*‐driven early gastric inflammation *via* the inflammasome, specifically through regulation of apoptosis and proliferation of gastric epithelial and immune cells.

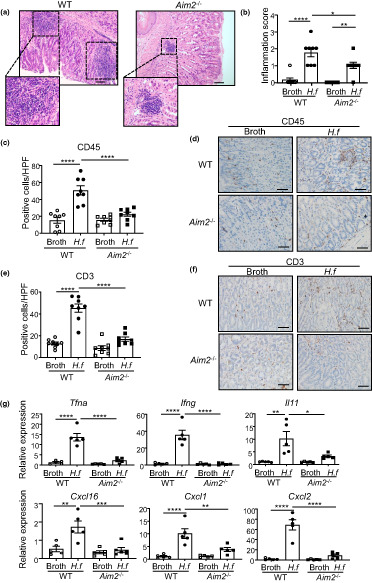

## INTRODUCTION

*Helicobacter pylori* (*H. pylori*) is a Gram‐negative bacterium which colonizes the stomach and is present in about 50% of the human population. *Helicobacter* infection in the gastric mucosa causes inflammation which, if dysregulated, can lead to the progression of atrophy, metaplasia, dysplasia and eventually intestinal‐type gastric adenocarcinoma, the major histopathological type of gastric cancer (GC), or mucosa‐associated lymphoid tissue lymphoma.^1^, ^2^, ^3^ Indeed, 1–3% of *H. pylori*–positive patients develop intestinal‐type GC, with about 10% of infected individuals suffering from other clinical complications including peptic ulcers.^4^, ^5^ The pathogen–host interaction of *Helicobacter* with the gastric mucosal immune system induces proinflammatory cytokine and reactive oxygen species release, resulting in pathogenic changes to cellular proliferation and homeostasis of the gastric epithelium, and recruitment and migration of immune cells within the stromal compartment.^6^, ^7^, ^8^, ^9^, ^10^, ^11^, ^12^ However, the molecular basis by which the innate immune system recognizes *Helicobacter* to drive these pathogenic processes is complex and not well understood.

Pattern recognition receptors (PRRs) are a superfamily of regulatory innate immune receptors which can have pro‐ and anti‐inflammatory functions.^13^ Among PRRs, the subfamily of Toll‐like receptors is the most widely investigated with regard to *Helicobacter* infection.^14^, ^15^, ^16^, ^17^, ^18^ Recently, we reported that the endosomal DNA sensor Toll‐like receptor 9 is upregulated in both patients with gastritis and GC, and promotes inflammation and proliferation of the gastric mucosal epithelium in a *Helicobacter* infection mouse model.^18^ Toll‐like receptor 2 has also been shown to recognize *Helicobacter* to activate several signaling pathways and interestingly, was implicated in promoting GC in an inflammation‐independent manner.^17^, ^19^ Nod‐like receptors (NLRs), in particular nucleotide‐binding oligomerization domain‐containing protein 1 (NOD1), nucleotide‐binding oligomerization domain‐containing protein 2 (NOD2) and NLR family pyrin domain containing 3 (NLRP3), the latter *via* inflammasome complexes and the interleukin (IL)‐1β effector cytokine, have also been reported to contribute to the *Helicobacter‐*induced pathogenic inflammatory response involving interferon‐gamma production.^10^, ^20^ A role for inflammasome‐associated inflammation in response to *Helicobacter* was supported through genetic ablation of the inflammasome adaptor, apoptosis‐associated speck‐like protein containing a CARD (ASC), which reduced inflammation and cytokine release in infected mice.^21^ In addition, genetic modulation of IL‐1β expression in knockout and transgenic mice has marked effects on *Helicobacter*‐induced apoptosis and immune cell infiltration in the gastric mucosal epithelium of mice, suggesting a major role for this inflammasome‐associated effector cytokine in *Helicobacter‐*induced gastric disease pathogenesis.^22^, ^23^ We also note that another inflammasome effector cytokine, IL‐18, was found to underpin inflammasome‐driven gastric tumorigenesis *via* suppression of apoptosis, independent of inflammation, and may also contribute to early gastric disease pathogenesis.^24^ Despite these observations, the full spectrum of PRRs that drive *Helicobacter* pathology remains elusive.

Absent in melanoma 2 (AIM2) is an innate immune cytosolic DNA sensor best known as an inflammasome‐associated PRR, and has been implicated in numerous inflammatory diseases and cancers.^13^, ^25^ Notably, AIM2 exhibits diverse cellular activities which invariably align with its context‐dependent cell and tissue expression, as well as its dual capacity to function both dependent and independent of the inflammasome.^13^, ^25^, ^26^, ^27^ Importantly, a recent study showed that transcriptional upregulation of *Aim2* by the signal transducer and activator of transcription 3 (STAT3) latent oncogenic transcription factor promoted GC *via* a direct, inflammasome‐independent role on epithelial cell migration.^28^ Conversely, AIM2 was recently implicated in downregulating T‐cell–mediated gastritis following *Helicobacter* infection, with the authors suggesting that this was independent of inflammasomes, despite classical read‐outs for inflammasome activation (e.g. caspase‐1 cleavage) not being assessed.^29^ Therefore, whether AIM2 exhibits a potential inflammasome‐dependent role in gastric disease pathogenesis remains unclear.

Here, by coupling *bona fide Aim2* knockout mice with a chronic *Helicobacter* infection model, together with clinical biopsies from infected patients, we reveal a driving role for AIM2 in *Helicobacter*‐induced gastric pathology. Specifically, AIM2 contributed to augmented proliferation and apoptosis of both gastric epithelial and immune cells upon *Helicobacter* infection. In addition, we show that, mechanistically, AIM2 functions *via* the inflammasome to release the IL‐1β effector cytokine. These findings help to unravel the disease‐stage dependent and complex roles of AIM2 in gastric disease.

## RESULTS

### *Helicobacter* infection upregulates AIM2 expression in human and mouse gastric tissue

**Figure 1:**
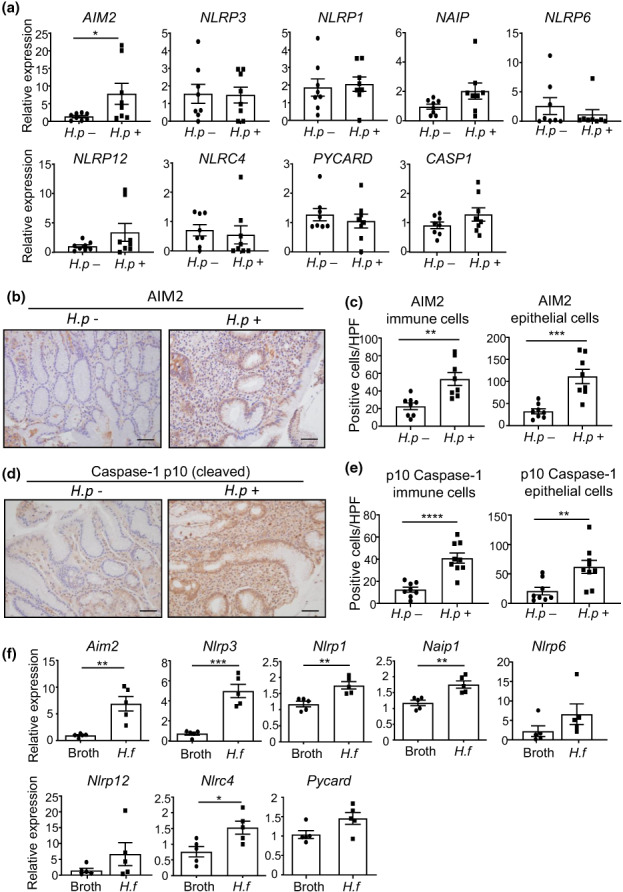
AIM2 expression is increased in gastric biopsies from individuals with *Helicobacter pylori* infection and stomachs of *Helicobacter felis*–infected mice. **(a)** Quantitative real‐time PCR expression analysis of PRRs and inflammasome components from *H. pylori*–positive and *H. pylori*–negative patients (*n* = 8/group). *P < 0.05; Mann–Whitney *U*‐test. Expression data are normalized to the human 18S ribosomal RNA housekeeping gene *RNA18S1*. **(b)** Representative images (40× magnification) of AIM2 immunohistochemistry of the indicated patient gastric biopsies. Scale bars = 50 μm. **(c)** Quantification of AIM2‐positive immunostained immune and epithelial cells in patient biopsies (*n* = 8/group). **P < 0.01, ***P < 0.001; Mann–Whitney *U*‐test. **(d)** Representative images (40× magnification) of cleaved caspase‐1 immunohistochemistry of patient gastric biopsies. Scale bars = 50 μm. **(e)** Quantification of cleaved (p10) caspase‐1‐positive immunostained immune and epithelial cells in patient biopsies (*n* = 8/group). **P < 0.01, ****P < 0.0001; Mann–Whitney *U*‐test. (**f**) Quantitative real‐time PCR expression analysis for PRRs and inflammasome components in gastric tissue of mice gavaged with *H. felis* or control broth (*n* = 5/group). *P < 0.05, **P < 0.01, ***P < 0.001; Student's *t*‐test. Expression data are normalized to the mouse 18S rRNA housekeeping gene *Rn18s*. All data were generated using three technical replicates (i.e. triplicates) from a composite of multiple independent experiments. AIM2, absent in melanoma 2; CASP1, caspase 1; *H.f*, *H. felis*; *H.p*, *H. pylori*; HPF, high‐power field; NAIP, NLR family apoptosis inhibitory protein; NLRC4, NLR family CARD domain containing 4; NLRP1, NLR family pyrin domain containing 1; NLRP3, NLR family pyrin domain containing 3; PRR, pattern recognition receptor; PYCARD, PYD and CARD domain containing.

To investigate a potential role for AIM2 in the initiating *H. pylori*–driven gastric inflammation stage of gastric carcinogenesis, we first measured *AIM2* gene expression levels by quantitative real‐time PCR (qPCR) in patients with gastritis with and without *Helicobacter* infection. Among a range of inflammasome‐associated PRRs and the inflammasome adaptor ASCs (encoded by *PYCARD*), only *AIM2* messenger RNA levels were significantly increased (~6‐fold) in *H. pylori*–positive *versus H. pylori*–negative gastritis patient biopsies, with *AIM2* also displaying the highest relative expression (Figure 1a). Consistently, immunohistochemical staining for AIM2 revealed more widespread and intense AIM2 positivity in gastric epithelial glandular tissue and immune cells within the lamina propria of *H. pylori*–positive samples than *H. pylori*–negative samples (Figure 1b, c). Considering the documented function of AIM2 in inflammasome complexes, we determined whether levels of caspase‐1 cleavage, a key indicator of inflammasome activity, was consistent with *Helicobacter* infection status in human gastric biopsies. Indeed, a significant increase in cleaved caspase‐1 immunostaining in epithelial and immune cells of *H. pylori*–positive samples was observed, indicating a higher level of inflammasome activity (Figure 1d, e).

To further investigate the effect of *Helicobacter* infection on PRR gene expression, we used a well‐established *Helicobacter felis* infection mouse model that reproducibly mimics chronic gastritis and gastric epithelial hyperplasia of *H. pylori* infection in human stomachs.^30^ At 4 months following oral gavage with *H. felis*, messenger RNA levels of *Aim2*, along with several other PRRs (*Nlrp3*, *Nlrp1*, *Naip1* and *Nlrc4*), were also elevated in stomachs of *H. felis*–infected mice compared with broth‐gavaged control mice, with relative *Aim2* expression the highest (Figure 1f). Taken together, these data suggest that AIM2 is highly upregulated in the stomach among inflammasome‐associated PRRs during *Helicobacter* infection.

### AIM2 promotes gastric inflammation and immune cell infiltrates in response to *Helicobacter* infection

**Figure 2:**
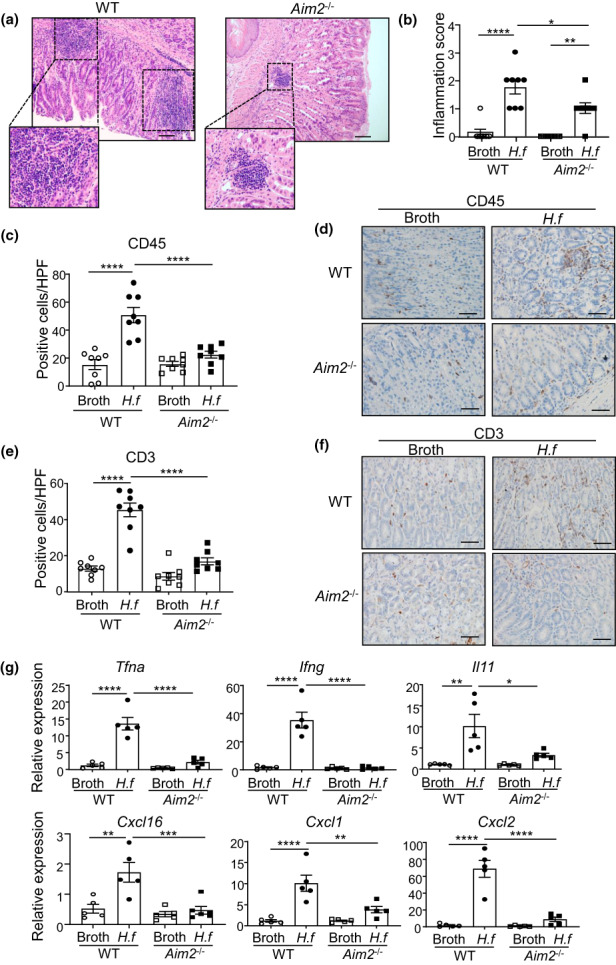
Suppressed inflammation and immune cell infiltrates in stomachs of *Aim2*‐deficient *Helicobacter felis*–infected mice. **(a)** Representative hematoxylin and eosin–stained *H. felis*–infected WT and *Aim2* ^−/−^ mouse stomachs taken at 20× magnification showing immune cell infiltrates. Scale bars = 100 μm. **(b)** Gastric inflammatory scores (0–3; none, mild, moderate, severe) of WT and *Aim2* ^−/−^ mice gavaged with *H. felis* or control broth (*n* = 8/group). *P < 0.05, **P < 0.01, ****P < 0.0001; one‐way ANOVA with multiple comparisons. **(c, e)** Quantification of CD45‐positive (**c**) and CD3‐positive (**e**) stained cells in WT and *Aim2* ^*−/−*^ *H. felis*– and broth‐gavaged mice (*n* = 8/group). ****P < 0.0001; one‐way ANOVA with multiple comparisons. **(d, f)** Representative images of CD45 **(d)** and CD3 **(f)** immunostaining in mouse gastric tissue cross‐sections from WT and *Aim2* ^*−/−*^ *H. felis*– and broth‐gavaged mice. Scale bars = 50 μm. **(g)** Quantitative real‐time PCR expression analysis of proinflammatory cytokines in *H. felis*– or broth‐gavaged WT and *Aim2* ^*−/−*^ mouse gastric tissues (*n* = 5/group). *P < 0.05, **P < 0.01, ***P < 0.0001, ****P < 0.0001; one‐way ANOVA with multiple comparisons. All data were generated using three technical replicates (i.e. triplicates) from a composite of multiple independent experiments. AIM2, absent in melanoma 2; *H.f*, *H. felis*; WT, wild type.

To determine a functional role for AIM2 in *Helicobacter*‐induced gastric pathology, we coupled *Aim2*‐deficient mice with the chronic *H. felis* infection model. In *H. felis*–infected *Aim2* ^*−/−*^ mice, histological scoring of hematoxylin and eosin–stained mouse stomach sections showed lower levels of inflammatory cell infiltrates compared with infected wild‐type (WT) mice, with the gastric inflammation score similarly reduced in both male and female *H. felis*–infected *Aim2* ^*−/−*^ mice (Figure 2a, b, Supplementary figure 1a). Furthermore, immunohistochemistry revealed a significant decrease (55.67%) in leukocyte/immune cell (CD45^+^) numbers in *H. felis*–infected *Aim2* ^*−/−*^ stomachs compared with infected WT counterparts (Figure 2c, d). Additionally, the increased numbers of T‐cell (CD3^+^) and B‐cell (B220^+^) infiltrates seen in *H. felis*–infected WT mouse stomachs were markedly reduced in the infected stomachs of *Aim2* ^*−/−*^ mice for T cells (by 63.34%) and B cells (by 56.72%; Figure 2e, f, Supplementary figure 1b–e). These observations were also supported at the molecular level, whereby qPCR indicated that the increased gastric gene expression levels of a range of proinflammatory cytokines and chemokines in *H. felis*–infected WT mice (compared with uninfected broth‐gavaged WT controls) were significantly reduced in *H. felis*–infected *Aim2* ^*−/−*^ mice to levels seen in uninfected WT mice (Figure 2g). These data therefore strongly suggest that AIM2 deficiency protects mice against *Helicobacter*‐induced pathologic inflammatory responses in the stomach.

### AIM2 promotes *Helicobacter*‐driven gastric epithelial and immune cell proliferation and apoptosis

**Figure 3:**
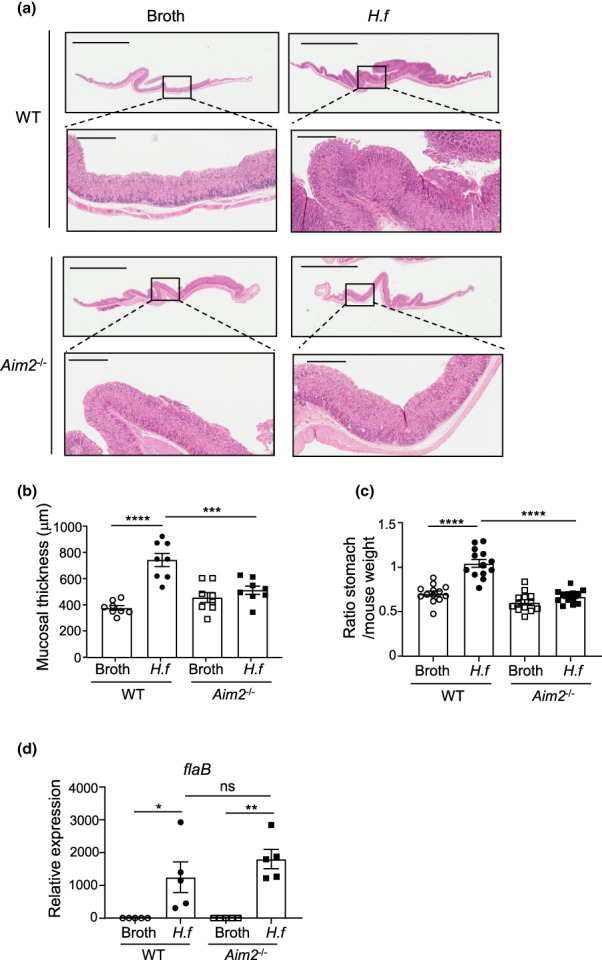
Severity of gastric epithelial hyperplasia is reduced in *Aim2* ^*−/−*^ *versus* WT *Helicobacter felis*–gavaged mice. **(a)** Representative images of hematoxylin and eosin–stained WT and *Aim2* ^*−/−*^ mouse gastric tissue cross sections showing mucosal thickness at 4 months postgavage with *H. felis* or control broth at 0.5× magnification (top panels, scale bars = 5 mm) and 4× magnification (bottom panels, scale bars = 500 μm). **(b)** Quantification of mucosal thickness of *H. felis* and control broth–gavaged WT and *Aim2* ^−/−^ mouse gastric corpus (*n* = 8/group). ***P < 0.001, ****P < 0.0001; one‐way ANOVA with multiple comparisons. **(c)** Ratio of stomach weight to total mouse weight for *H. felis–* and control broth–gavaged WT and *Aim2* ^−/−^ mice (*n* = 13/group). ****P < 0.0001; one‐way ANOVA with multiple comparisons. **(d)** *H. felis* colonization of corpus tissue determined by quantitative real‐time PCR gene expression for *H. felis*–specific *flaB* (relative to mouse *18S*) in genomic DNA of stomachs from the indicated WT and *Aim2* ^−/−^ mouse groups (*n* = 5/group). All data were generated using three technical replicates (i.e. triplicates) from a composite of multiple independent experiments. *P < 0.05; **P < 0.01; one‐way ANOVA with multiple comparisons. ns, not significant. AIM2, absent in melanoma 2; *H.f*, *H. felis*; WT, wild type.

We next evaluated whether AIM2 also contributed to gastric epithelial hyperplasia, evidenced by augmented mucosal thickness of the gastric corpus, that is a feature of chronic *Helicobacter* infection.^31^ Notably, while *H. felis*–infected WT mice exhibited marked increases in stomach weights (56.75%) and corpus mucosal thickness (96.97%) compared with broth control mice, the stomach weights and corpus mucosal thickness of infected *Aim2* ^*−/−*^ mice were significantly reduced by 35.90% and 31.07%, respectively, compared with infected WT mice (Figure 3a–c). These observed reductions in stomach weights and corpus mucosal thickness of infected *Aim2* ^*−/−*^ *versus* WT mice were similar among both males and females, suggesting that AIM2 promotes gastric hyperplasia irrespective of gender (Supplementary figure 2a, b). The suppressed *H. felis*–driven gastric pathology in *Aim2* ^*−/−*^ mice was not because of the inability of *H. felis* to colonize mouse stomachs, as qPCR expression analysis of the *H. felis*–specific *flaB* gene showed that the gastric corpus bacterial loads of *H. felis* were similarly increased in both WT and *Aim2* ^*−/−*^ infected mice compared with their uninfected counterparts (Figure 3d).

**Figure 4:**
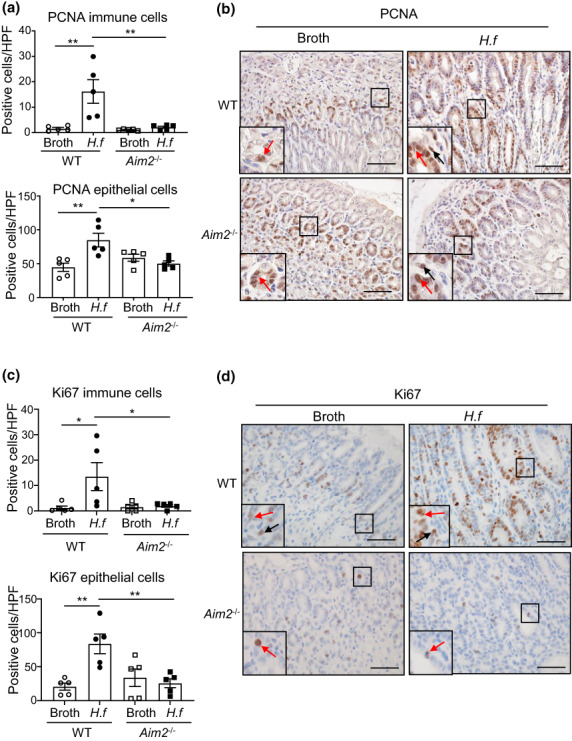
AIM2 promotes proliferation in response to *Helicobacter felis* infection in mouse stomachs **(a, c)**. Quantification of PCNA **(a)** and Ki67 **(c)** immunostained immune cells (top graph) and epithelial cells (bottom graph) in mouse gastric tissue (*n* = 5/group). *P < 0.05, **P < 0.01; one‐way ANOVA with multiple comparisons. **(b, d)** Representative images of **(b)** PCNA and **(d)** Ki67 immunostaining in mouse gastric tissue. Insets at the bottom left of each image depict magnified areas (open squares) in the main images. Black arrows depict positively stained immune cells, and red arrows depict positively stained epithelial cells, in broth and *H. felis*–gavaged WT and *Aim2* ^−/−^ mouse stomach sections. Scale bars = 50 μm. All data were generated using three technical replicates (i.e. triplicates) from a composite of multiple independent experiments. AIM2, absent in melanoma 2; *H.f*, *H. felis*; HPF, high‐power field; PCNA, proliferating cell nuclear antigen.

In addition, immunohistochemical staining for markers of cellular proliferation, proliferating cell nuclear antigen (PCNA) and Ki67, revealed a significant increase in the numbers of positive cells in both gastric epithelial and immune compartments of *H. felis*–infected WT *versus Aim2* ^*−/−*^ mice (Figure 4a–d). Importantly, immunofluorescence costaining of PCNA with the epithelial marker, E‐cadherin, and the pan‐immune cell marker, CD45, confirmed pronounced colocalization of PCNA positivity in both epithelial and immune cells of *H. felis*–infected WT mice compared with *Aim2* ^*−/−*^ mice (Supplementary figure 3a, b). These data therefore support a role for AIM2 in driving gastric epithelial and immune cellular proliferation in response to *Helicobacter* infection.

**Figure 5:**
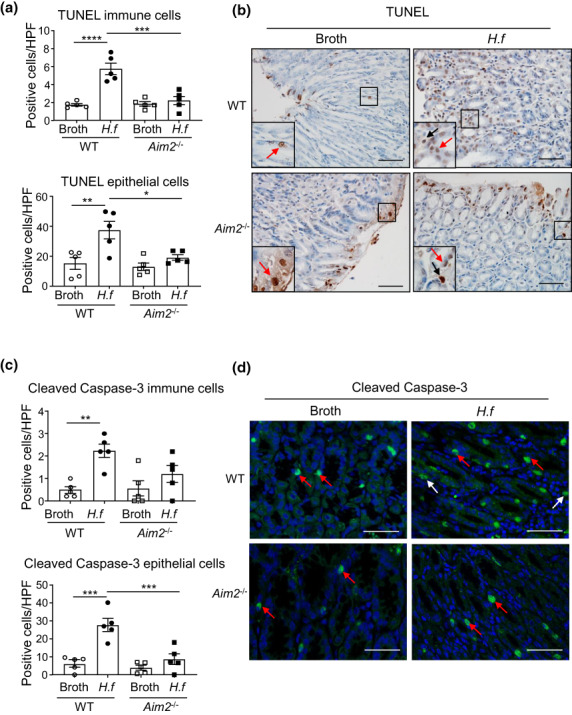
*Aim2* promotes apoptosis in response to *Helicobacter felis* infection in mouse stomachs. **(a)** Quantification of TUNEL‐immunostained immune cells (top graph) and epithelial cells (bottom graph) in mouse gastric tissue cross sections (*n* = 5/group). *P < 0.05, **P < 0.01, ***P < 0.001, ****P < 0.0001; one‐way ANOVA with multiple comparisons. **(b)** Representative images of TUNEL immunostaining in mouse gastric tissue cross sections. Insets at the bottom left of each image depict magnified areas (open squares) in the main images. In insets, black arrows depict positively stained immune cells, and red arrows depict positively stained epithelial cells, in broth‐ and *H. felis*–gavaged WT and *Aim2* ^−/−^ mouse stomach sections. Scale bars = 50 μm. **(c)** Quantification of cleaved caspase‐3 immunofluorescence‐positive immune cells and epithelial cells in mouse gastric tissue cross sections (*n* = 5/group). **P < 0.01, ***P < 0.001; one‐way ANOVA with multiple comparisons. **(d)** Representative images of cleaved caspase‐3 immunofluorescence (green) plus DAPI nuclear staining (blue) in mouse gastric tissue cross sections. Red arrows depict positively stained epithelial cells and white arrows depict positively stained immune cells. Scale bars = 50 μm. All data were generated using three technical replicates (i.e. triplicates) from a composite of multiple independent experiments. AIM2, absent in melanoma 2; DAPI, 4′,6‐diamidino‐2‐phenylindole; *H.f*, *H. felis*; HPF, high‐power field; TUNEL, terminal deoxynucleotidyl transferase dUTP nick end labeling; WT, wild type.

We next evaluated the effect of *Helicobacter* infection on gastric cellular apoptosis by performing immunohistochemistry and immunofluorescence analyses for markers of apoptosis, TUNEL (terminal deoxynucleotidyl transferase dUTP nick end labeling) and cleaved caspase‐3, respectively. Indeed, the increased numbers of TUNEL‐positive apoptotic cells observed in *H. felis*–infected WT mouse stomachs (compared with broth WT controls) were significantly reduced in *H. felis*–infected *Aim2* ^*−/−*^ mice in both gastric epithelial cells (by 48.93%) and immune cell infiltrates (by 61.39%) (Figure 5a, b). Similarly, immunofluorescence indicated a 68.54% and 46.43% reduction in the numbers of cleaved caspase‐3 positively stained apoptotic gastric epithelial cells and immune cell infiltrates, respectively, in the stomachs of *H. felis*–infected *Aim2* ^*−/−*^ mice *versus* WT mice (Figure 5c, d). Collectively, the suppressed pathologic proliferative and apoptotic epithelial and immune cellular responses in the gastric compartment of *Helicobacter*‐infected *Aim2* ^*−/−*^ mice suggest that AIM2 contributes to gastric pathology *via* modulating these cellular functions throughout the inflamed gastric mucosa.

### AIM2 functions *via* the inflammasome to promote gastric pathology during *Helicobacter* infection

**Figure 6:**
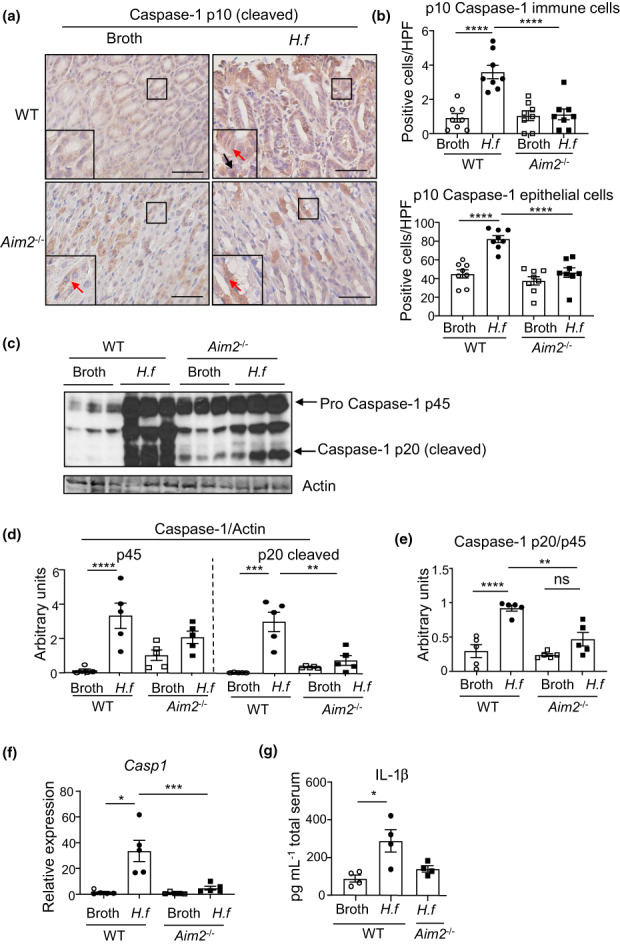
Suppressed inflammation in *Aim2* ^*−/−*^ *Helicobacter felis* mouse stomachs correlates with reduced inflammasome activity. **(a)** Representative images of cleaved caspase‐1 immunostaining in gastric tissue sections from broth‐ and *H. felis*–gavaged WT and *Aim2* ^−/−^ mice. Red arrows depict positively stained epithelial cells and black arrows depict positively stained immune cells. Scale bars = 50 μm. **(b)** Quantification of positively immunostained cleaved (p10) caspase‐1 immune cells (top graph) and epithelial cells (bottom graph) in the indicated mouse gastric tissue groups (*n* = 8/group). ****P < 0.0001; one‐way ANOVA with multiple comparisons. **(c)** Immunoblot for caspase‐1 showing cleaved (p20) and pro (p45) forms of the protein indicated with arrows. Each lane represents an individual mouse sample. Actin is used as a loading control. **(d, e)** Semiquantitative densitometry of the relative expression of cleaved and pro forms of caspase‐1 normalized to actin **(d)**, and cleaved caspase‐1 normalized to procaspase‐1 **(e)** (*n* = 5/group). **P < 0.01, ***P < 0.001, ****P < 0.0001; one‐way ANOVA with multiple comparisons. ns, not significant. **(f)** Quantitative real‐time PCR expression analysis for *Casp1* in *H. felis–* or broth‐gavaged WT and *Aim2* ^*−/−*^ mouse gastric tissues (*n* = 5/group). *P < 0.05, ***P < 0.001; one‐way ANOVA with multiple comparisons. **(g)** ELISA for IL‐1β on *H. felis*– *versus* broth‐gavaged WT, and *H. felis*–gavaged *Aim2* ^−/−^, mouse serum (*n* = 4/group). *P < 0.05; Student's *t*‐test. All data were generated using three technical replicates (i.e. triplicates) from a composite of multiple independent experiments. AIM2, absent in melanoma 2; *H.f*, *H. felis*; IL, interleukin; WT, wild type.

Considering AIM2 has a well‐known function in the formation of inflammasome complexes, we next assessed whether AIM2 deficiency would impair the activation levels of the key inflammasome effector, caspase‐1, during chronic *Helicobacter* infection. Indeed, immunohistochemistry revealed there were higher levels of cellular (immune and epithelial) staining for the mature, cleaved (i.e. activated) form of caspase‐1 (p10) in gastric tissue cross sections from *H. felis*–infected WT compared with *Aim2* ^*−/−*^ mice, irrespective of gender (Figure 6a, b, Supplementary figure 4a, b). In addition, immunoblotting together with densitometric quantification for caspase‐1 protein expression showed increased amounts of the cleavage product (p20), as well as the immature proform (p45), of caspase‐1, in gastric tissue lysates of *H. felis*–infected WT mice compared with *H. felis*–infected *Aim2* ^*−/−*^ mice (Figure 6c–e). Interestingly, while basal gastric levels of p20 and p45 caspase‐1 forms appeared elevated in broth control *Aim2* ^*−/−*^ compared with WT mice, this did not equate to an increase in the relative cleavage of the p45 proform to the mature/activated p20 form (based on the p20/p45 ratio; Figure 6c–e). In addition, messenger RNA levels of caspase‐1 were diminished in *H. felis*–infected *Aim2* ^*−/−*^ mice compared with *H. felis*–infected WT mice (Figure 6f). These findings suggesting that AIM2 is associated with inflammasome complexes during chronic *Helicobacter* infection were further supported by our observations that secreted circulating protein levels of the bioactive form of the inflammasome effector cytokine, IL‐1β, were elevated in serum of *H. felis*–infected WT mice compared with broth controls, while serum IL‐1β protein levels were decreased in *H. felis*–infected *Aim2* ^*−/−*^ mice, as measured by ELISA (Figure 6g). By contrast, serum protein levels of the other major inflammasome effector cytokine, IL‐18, were undetectable by ELISA (data not shown). Overall, these data support our findings of *Helicobacter*‐associated elevated inflammasome activity in human gastric tissue biopsies, and suggest that AIM2 *via* inflammasomes promotes *Helicobacter*‐induced gastric disease.

## DISCUSSION

The host immune response to *Helicobacter* infection in the stomach is a critical determinant in the extent and severity of gastric inflammation and subsequent risk of GC. While members of the innate immune Toll‐like receptor and NLR families have been extensively investigated in the early stages of *Helicobacter* infection and gastric disease, here we focused on the cytosolic DNA sensor, AIM2. Our investigation revealed that AIM2 was critical for promoting *Helicobacter*‐induced chronic gastric pathology comprising inflammation and epithelial hyperplasia, *via* altering proliferation and apoptosis of gastric epithelial and immune cells, recruitment of immune cells and production of proinflammatory cytokines and chemokines.

A key finding of our study was that AIM2 messenger RNA and protein levels were upregulated in *H. pylori*–positive human gastric biopsies and gastric tissues from *H. felis–*infected mice, consistent with inflammasome activity. Interestingly, this contrasts recent findings that other DNA sensors, namely, stimulator of interferon genes (STING) and retinoid acid inducible gene I (RIG‐I), are downregulated by *Helicobacter* in an 8‐week infection mouse model where deletion of *Sting1* or *Rigi* reduced acute immune responses but not chronic gastric inflammation.^32^ The expression of both these genes also negatively correlates with GC prognosis in patients.^32^, ^33^, ^34^ This contrasts that of AIM2, whose upregulated expression in patients with GC correlates with impaired survival.^28^ Furthermore, in a hyperinflammatory mouse model of spontaneous GC driven by deregulated cytokine signaling *via* the IL‐11/STAT3 axis, we recently reported that AIM2 promotes the progression of GC independent of the inflammasome and inflammation, but rather by augmenting intrinsic epithelial cell migration.^28^ While this contrasts our current findings that the AIM2 inflammasome promotes *Helicobacter*‐driven gastric inflammation, these findings nonetheless suggest a dichotomous role for AIM2 in which it promotes early stage *Helicobacter* infection–driven gastritis in an inflammasome‐dependent manner, and late‐stage cytokine–driven gastric tumorigenesis independent of inflammasomes. A possible explanation for the divergent functions of AIM2 in gastric disease is the cell‐type context of its expression. Indeed, in GC, AIM2 upregulation was tumor intrinsic and restricted to the gastric epithelium, while we demonstrate here that AIM2 is upregulated in both gastric epithelial and immune cells in early stage *Helicobacter*‐related gastritis.^28^ We also note that an inflammasome‐independent role for AIM2 that is intrinsic to the gut epithelium has been demonstrated in colon cancer, whereby AIM2 suppresses intestinal tumorigenesis in Apc^Min/+^ and DSS/AOM mouse models by modulating intestinal epithelial cell apoptosis and proliferation.^25^, ^35^ While these observations suggest that inflammasome‐independent roles for AIM2 in gastrointestinal pathologies predominantly align with AIM2 expression and function in the epithelium, it is also noteworthy that AIM2 inflammasome activity intrinsic to the gut epithelium has been attributed to the regulation of intestinal homeostasis in mouse models of colitis in both epithelial and macrophages, specifically *via* IL‐18 production.^27^, ^36^ Therefore, the complex context dependency of AIM2 pathophysiological functions and molecular mechanisms (i.e. inflammasome dependent *versus* independent) in the gastrointestinal tract are likely underpinned by not only its expression in specific cell types (i.e. epithelial *versus* immune), but also tissue types and disease stage, as well as the type (i.e. endogenous *versus* microbial) and subcellular location (i.e. cytosol *versus* nucleus) of activating ligands. Importantly, considering these complexities regarding AIM2 biological functions in gastrointestinal pathologies, future studies are warranted to couple conditional knockout strains of AIM2 with gastrointestinal disease models (including *Helicobacter* infection) to identify specific AIM2‐expressing cell types that modulate disease pathogenesis.

We also observed that AIM2 promoted proliferation and apoptosis in both gastric epithelial and immune cells, which suggests that disrupted cellular homeostasis may be a key mechanism underlying *Helicobacter*‐related pathology. Dysregulated epithelial cell proliferation is often associated with inflammation throughout the gastrointestinal system and it has been documented that *Helicobacter* alters both proliferation and apoptosis of gastric cells.^37^, ^38^ We speculate that dysregulation of epithelial cell homeostasis by upregulation of AIM2 results in loss of barrier integrity which exacerbates the inflammatory response as the underlying tissue is more exposed to *Helicobacter* and other pathogens. The role of AIM2 in barrier function has been reported in the context of *Salmonella* infection in the colon, albeit with its expression having an opposing effect (i.e. promoting barrier integrity).^39^ The differences in AIM2 on gastric and intestinal disease further exemplify the diverse roles of AIM2 and suggest that the gastrointestinal tract may be finely tuned to a physiological threshold of AIM2 expression/activation for homeostasis whereby disrupting this balance compromises integrity.

We also refer our current findings to a recent study suggesting that AIM2 independent of inflammasomes has a protective role against *Helicobacter*‐induced gastric inflammation and associated spasmolytic peptide‐expressing metaplasia. Specifically, using a 6‐month *H. felis* infection spasmolytic peptide‐expressing metaplasia mouse model, AIM2 was proposed to selectively suppress the recruitment of interferon‐gamma–producing gastric CD8^+^ T cells (along with impaired expression of key T‐cell homing receptors; e.g. Selectin L, Sphingosine‐1‐Phosphate Receptor 1) *via* a B‐cell–intrinsic function that blocked production of the T‐cell chemoattractant, chemokine (C–X–C motif) ligand 16.^29^ These observations contrast our study, where we also did not observe any changes in expression of either interferon‐gamma, chemokine (C–X–C motif) ligand 16 or T‐cell homing receptors (Supplementary figure 5) in the stomachs of infected *Aim2* ^−/−^ mice. In considering an explanation for the opposing observations of these two studies, it is important to note that El‐Zaatari and colleagues employed *Aim2* gene‐trap mice, which express residual AIM2, unlike our current study that employed *bona fide* whole‐body AIM2‐deficient mice.^28^, ^29^, ^40^, ^41^ Furthermore, the suggestion that AIM2 acted independent of inflammasomes was limited to assessment of only (unaltered) protein levels of IL‐18 and IL‐1β secreted from *ex vivo* gastric explants of WT and *Aim2* gene‐trap mice, with no *in vivo* assessment of protein levels of mature forms of these inflammasome effector cytokines, nor levels of cleaved (i.e. active) caspase‐1, a hallmark of AIM2 inflammasome activity.^29^ We also note the possibility that these studies, which employ *H. felis* infections over different periods (i.e. 4 months *versus* 6 months), may further uncover multiple, and potentially contrasting, roles for AIM2 during various stages (e.g. gastritis, metaplasia) of chronic *Helicobacter* infection. In this respect, in addition to AIM2, several other inflammasome‐associated components (*via* IL‐1β) that have been reported to contribute to gastric inflammation and pathology in *Helicobacter* infection mouse models, namely ASC and NLRP3,^10^, ^21^, ^42^ also play contrasting roles within a specific disease setting that are dependent on expression in specific cell types and stages of disease.^43^, ^44^, ^45^, ^46^

In summary, our current study suggests that the AIM2 inflammasome is a critical regulator of inflammation in the earlier stages of *Helicobacter*‐induced gastritis and associated epithelial hyperplasia. Coupled with the finding that AIM2 can also act independent of inflammasomes to promote later stage gastric adenocarcinoma, these observations expand our current understanding of the complexities of AIM2 function during the cascade of events and immunoregulatory checkpoints that underpin gastric carcinogenesis. Furthermore, these inflammasome‐dependent (early stage gastritis) and inflammasome‐independent (late stage gastric adenocarcinoma) mechanism(s) of action of AIM2 have potential to guide distinct therapeutic strategies against AIM2 at specific stages of the gastric carcinogenesis cascade.

## METHODS

### Patient biopsies

Gastric body samples were collected from patients with non‐GC undergoing endoscopy at Monash Medical Centre (Melbourne, VIC, Australia; Supplementary table 1). Exclusion criteria included individuals with serious concomitant illness, observable tumors, gastrointestinal bleeding, previous gastric surgeries and prior use of proton pump inhibitors, antibiotics or nonsteroidal anti‐inflammatory drugs. Biopsies were either snap‐frozen in liquid nitrogen or stored in 10% formalin, the latter for histopathology.^47^ The *H. pylori* status was determined by *16S* rRNA qPCR and assessment of Cresyl fast violet–stained tissue sections as described before.^31^ We note that the etiology of *H. pylori*–negative gastritis is poorly understood, and likely to involve overgrowth of other pathogenic microbes (e.g. *Streptococcus*).^48^, ^49^ Written informed consent was obtained from each patient, and biopsy collections were approved by the Monash Health Human Research Ethics Committee. Patient studies were conducted in accordance with the World Medical Association Declaration of Helsinki statement on the ethical principles for medical research involving human subjects.

### Mice

*Aim2*‐knockout (*Aim2* ^*−/−*^) mice on a mixed C57BL6 × 129Sv background have been previously described,^28^, ^40^ and genetically matched WT littermates were used as controls. Where possible, equal numbers of male and female mice were randomly assigned to experimental groups. All animals were housed under specific pathogen‐free conditions, and studies were approved by the Monash University Monash Medical Centre “B” Animal Ethics Committee.

### *In vivo H. felis* infection model

*Helicobacter felis* (strain CST1)^50^, ^51^ was cultured and harvested on horse blood agar or brain heart infusion broth. *H. felis* suspensions for mouse inoculations were prepared from bacteria on horse blood agar plates using brain heart infusion broth.^30^ Four‐ to six‐week‐old WT or *Aim2* ^*−/−*^ mice were administered with either control brain heart infusion broth or approximately 10^7^ *H. felis* bacterial suspension by single oral gavage (200 μL) using polyethylene catheters.^31^ Mice were culled at 4 months after infection for subsequent analyses. *H. felis* bacterial loads were confirmed using qPCR amplification of the *H. felis flab* gene as previously described.^31^

### Gene expression analyses

Equivalent amounts of total RNA were isolated from *Helicobacter*‐positive and *Helicobacter*‐negative snap‐frozen mouse (100 mg) and human (100 mg) stomach tissue biopsies using TRI Reagent Solution (Sigma‐Aldrich, St Louis, MO, USA), followed by on‐column RNeasy Mini Kit RNA clean‐up and DNase treatment (Qiagen, Hilden, Germany). Total RNA was then reverse transcribed (1 μg in 20 μL reaction volume for each RNA sample) with the Transcriptor High Fidelity cDNA Synthesis Kit (Sigma‐Aldrich). Each qPCR was performed on 2 μL diluted complementary DNA (diluted 1:10 for mouse, 1:5 for human samples) with SYBR Green chemistry (Life Technologies, Carlsbad, CA, USA) and a final primer concentration of 2 μM using the QuantStudio 6 Flex Real‐Time PCR System (Applied Biosystems, Waltham, MA, USA). Cycling conditions were as follows: initial denaturation, 95°C for 10 min; 40 cycles of denaturation (95°C for 15 s) and annealing (60°C for 1 min) with ramp rate of 1.6°C/s and a final melt curve step of 95°C for 15 s, 60°C for 1 min at 1.6°C/s and 95°C for 15 s with ramp rate of 0.05°C/s. Data acquisition and analyses were undertaken using the QuantStudio software version 1.3, including comparative CT (ΔΔCT) analyses. PCR product specificity was confirmed by melting curve analyses. Reverse transcriptase–negative complementary DNA samples and water were used in SYBR reactions as controls. Forward and reverse primer sequences for mouse and human primers are presented in Supplementary table 2.

### Immunostaining, immunofluorescence and histology

Following formalin fixation and paraffin embedding, histological assessment of mouse stomachs and patient gastric biopsies were performed on 4–6‐μm hematoxylin and eosin–stained tissue sections. Gastric mucosal thickness was measured as previously published.^18^, ^31^ Immunohistochemistry with primary antibodies against PCNA (ab18197; Abcam, Cambridge, UK), CD45 (550539; BD Biosciences, Franklin Lakes, NJ, USA), CD68 (ab125212; Abcam), CD3 (sc‐1127; Santa Cruz, TX, USA), AIM2 (HPA031365; Atlas Antibodies, Stockholm, Sweden) and Ki67 (16 667; Abcam) was performed on gastric tissue sections as described previously.^28^ For immunohistochemistry with a primary antibody against cleaved caspase‐1 (PA5‐105049; p10, cleaved caspase‐1 Ala317; Invitrogen, Waltham, MA, USA) on formalin fixation and paraffin embedding tissue sections, antigen retrieval was performed with ethylenediaminetetraacetic acid buffer (1 mM, pH 8.0), using a 1–2‐h block in CAS‐Block (Thermo Fisher, Waltham, MA, USA). Sections were counterstained with hematoxylin. Immunofluorescence staining for cleaved caspase‐3 (9661; Cell Signaling Technologies, Danvers, MA, USA), PCNA (ab18197; Abcam), E‐cadherin (3195P; Cell Signaling Technologies) and CD45 (550 539; BD Biosciences) was performed as described previously.^24^ Primary antibodies were detected with Alexa Fluor secondary antibodies and nuclear staining was performed with 4′,6‐diamidino‐2‐phenylindole. Individual positively stained cells were counted manually in a blinded manner by investigators using ImageJ analysis software (National Institutes of Health, Bethesda, MD, USA). The number of positively stained cells was counted per high‐power (×40) field, excluding areas containing lymphoid follicles, of 500 μm × 350 μm (immunohistochemistry) and 350 μm × 350 μm (immunofluorescence) (*n* = 5).

### Immunoblotting

Total protein lysates (25 μg) from snap‐frozen mouse tissues were prepared for immunoblotting with antibodies against mouse caspase‐1 (p45/p20) (AG‐20B‐0042‐C100; AdipoGen, San Diego, CA, USA) and mouse actin (A4700; Sigma‐Aldrich), as described previously.^28^ Blots were visualized using enhanced chemiluminescence for caspase‐1 or IR Dye 800 (Li‐COR Biosciences, Lincoln, NE, USA) for actin, and imaged with the Bio‐Rad ChemiDoc or the Odyssey Infrared Imaging System (LI‐COR). Densitometry analysis of bands was quantified using ImageJ software (*n* = 5).

### ELISA

IL‐1β ELISA was performed on mouse serum as per manufacturer's instructions (R&D Systems, Minneapolis, MN, USA). Mature IL‐18 ELISA was performed as described previously.^24^

### Statistical analyses

All statistical analyses were performed using GraphPad Prism V9 software. Statistical significance (*P* < 0.05) between the means of two groups was determined using Student's *t*‐test or the Mann–Whitney *U*‐test. Statistical significance between the means of multiple groups were determined using ordinary one‐way ANOVA with *post hoc* multiple comparisons pairwise tests. All data are presented as the mean ± standard error of the mean from at least three technical replicates. The log‐rank test was used to calculate the statistical significance of the difference in survival.

## AUTHOR CONTRIBUTIONS

**Ruby E Dawson:** Data curation; formal analysis; writing – original draft; writing – review and editing. **Virginie Deswaerte:** Data curation; formal analysis. **Alison C West:** Data curation. **Ekimei Sun:** Data curation; formal analysis. **Georgie Wray‐McCann:** Data curation. **Thaleia Livis:** Data curation. **Beena Kumar:** Formal analysis. **Emiliana Rodriguez:** Data curation. **Cem Gabay:** Formal analysis; resources; supervision. **Richard L Ferrero:** Resources; writing – review and editing. **Brendan J Jenkins:** Conceptualization; formal analysis; supervision; writing – original draft; writing – review and editing.

## CONFLICT OF INTEREST

All authors have no conflicts of interest to declare.

## FUNDING INFORMATION

This work was funded by a Peer Reviewed Cancer Research Program Idea Award (CA210128) from the United States Department of Defense (BJJ). This work was also supported by the Operational Infrastructure Support Program by the Victorian Government of Australia, and a Project Grant (APP1139371) from the National Health and Medical Research Council (NHMRC) of Australia (BJJ). BJJ and RLF were supported by NHMRC Senior Research Fellowships.

## Supporting information


Supplementary figure 1
Supplementary figure 2
Supplementary figure 3
Supplementary figure 4
Supplementary figure 5
Supplementary table 1
Supplementary table 2

## Data Availability

The data that support the findings of this study are available from the corresponding author upon reasonable request.
