# Supplementary material for: The cytosolic DNA sensor AIM2 promotes Helicobacter‐induced gastric pathology via the inflammasome
Source: Immunol Cell Biol. 2023 Apr 19;101(5):444–57. doi: 10.1111/imcb.12641 (PMC10952813; doi:10.1111/imcb.12641)
Supplement: Supplementary file 1 — Supplementary figure 1 Supplementary figure 2 Supplementary figure 3 Supplementary figure 4 Supplementary figure 5 Supplementary table 1 Supplementary table 2 [file IMCB-101-444-s001.docx]

**The cytosolic DNA sensor AIM2 promotes *Helicobacter-*induced gastric pathology via the inflammasome**

Ruby E Dawson, Virginie Deswaerte, Alison C West, Ekimei Sun, Georgie Wray-McCann, Thaleia Livis, Beena Kumar, Emiliana Rodriguez, Cem Gabay, Richard L Ferrero, Brendan J Jenkins

**Inventory of Supplementary Information**

**Supplementary figure 1:** Immune cell infiltrates in stomachs of WT and *Aim2*-deficient *H. felis*-infected mice.

**Supplementary figure 2**: Suppressed gastric pathology in *Aim2*-deficient *H. felis*-infected mice is independent of gender.

**Supplementary figure 3**: Co-localisation of proliferating PCNA-positive epithelial and immune cells in *H. felis*-infected mouse gastric tissues.

**Supplementary figure 4**: Reduced Caspase-1 activity in *Aim2^-/-^* *H. felis* mouse stomachs is independent of gender bias

**Supplementary figure 5:** Gene expression analysis of selected immune homing receptors in *H. felis*-infected mouse gastric tissues.

**Supplementary table 1:** Gastritis patient biopsies with indicated *Helicobacter pylori* status and score of inflammation severity.

**Supplementary table 2:** Primer sequences used for qPCR gene expression assays.

**
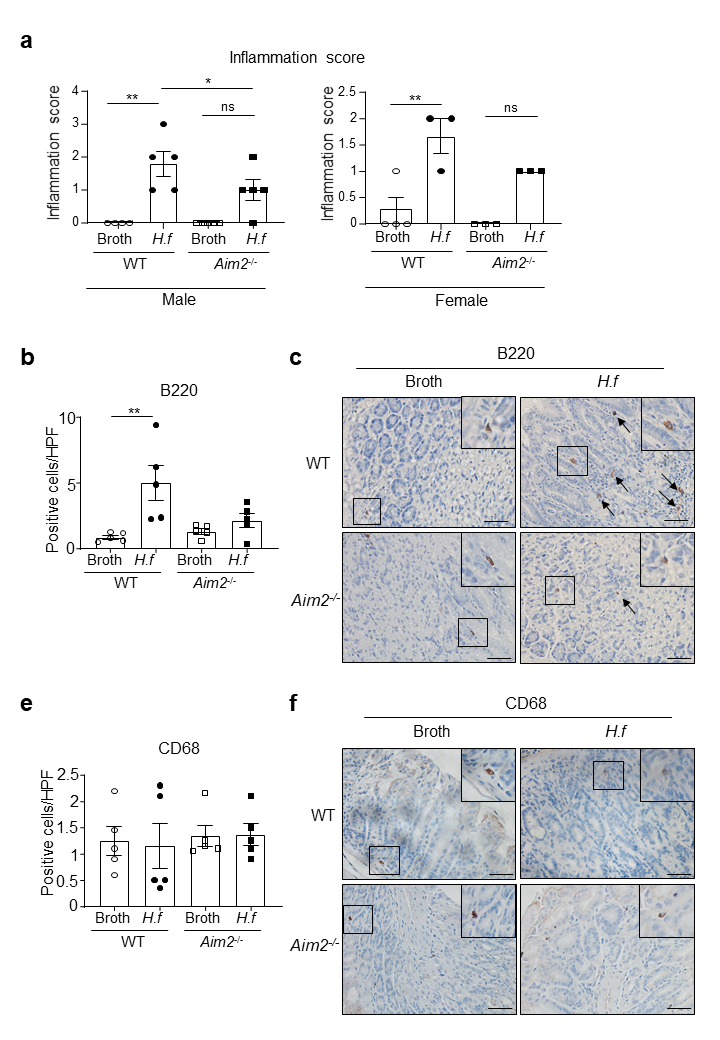
**

**Supplementary figure 1: Immune cell infiltrates in stomachs of WT and *Aim2*-deficient *H. felis*-infected mice. (a)** Gastric inflammatory scores (0–3; none, mild, moderate, severe) of segregated male and female WT and *Aim2*^-/-^ mice gavaged with *H. felis* or control broth (n = 3–5/group). **P* < 0.05, ***P* < 0.01; One-way ANOVA with multiple comparisons. ns, not significant. **(b)** Quantification of B220-positive stained cells in WT and *Aim2^-/-^* *H. felis* and broth gavaged mice (n = 5/group). ***P* < 0.01; One-way ANOVA with multiple comparisons. **(c)** Representative images of B220-positive immunostaining (arrows) in mouse gastric tissue cross-sections from WT and *Aim2^-/-^* *H. felis* and broth gavaged mice. Scale bars = 50 μm. Insets at the top right of each image depict magnified areas (open squares) in the main images containing a B220-positive B cell. **(d)** Quantification of CD68-positive stained cells in WT and *Aim2^-/-^* *H. felis* and broth gavaged mice (n = 5/group). **(e)** Representative images of CD68 immunostaining in mouse gastric tissue cross-sections from WT and *Aim2^-/-^* *H. felis* and broth gavaged mice. Scale bars = 50 μm. Insets at the top right of each image depict magnified areas (open squares) in the main images containing a CD68-positive macrophage.

**
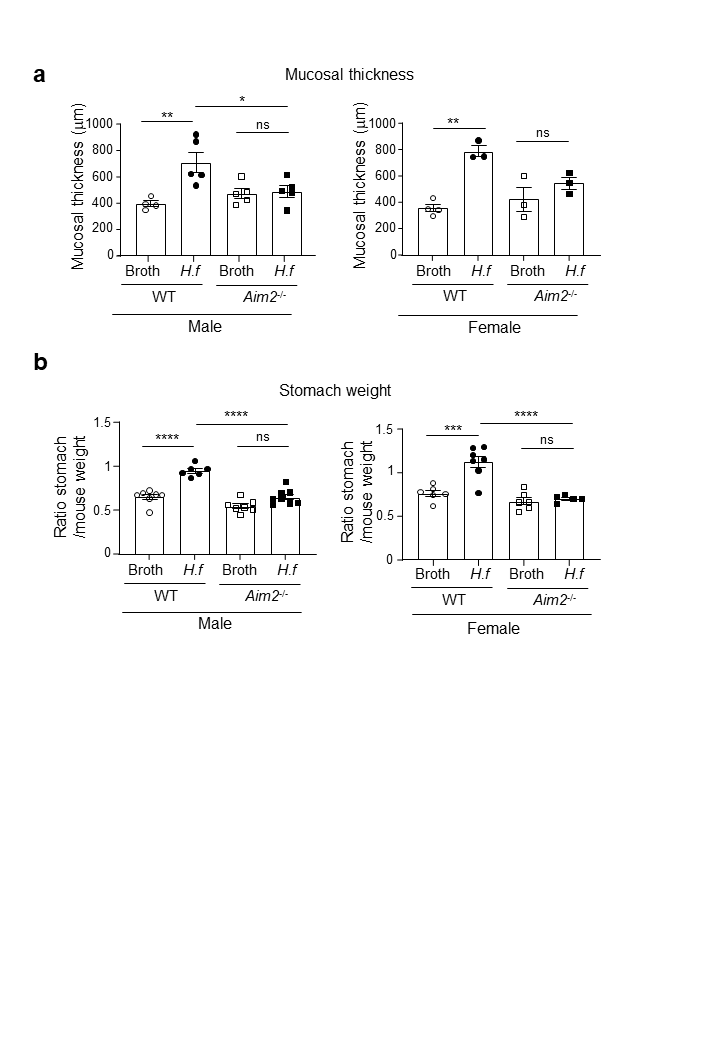
Supplementary figure 2**: Suppressed gastric pathology in *Aim2*-deficient *H. felis*-infected mice is independent of gender. **(a)** Quantification of mucosal thickness of *H. felis* and control broth gavaged WT and *Aim2*^-/-^ gastric corpus in mice segregated into males and females (n = 3-5/group). **P* < 0.05, ***P* < 0.01; One-way ANOVA with multiple comparisons. ns, not significant. **(b)** Ratio of stomach weight to total mouse weight for *H. felis* and control broth gavaged WT and *Aim2*^-/-^ mice segregated into males and females (n = 5–8/group). ****P* < 0.001, *****P* < 0.0001; One-way ANOVA with multiple comparisons. ns, not significant.


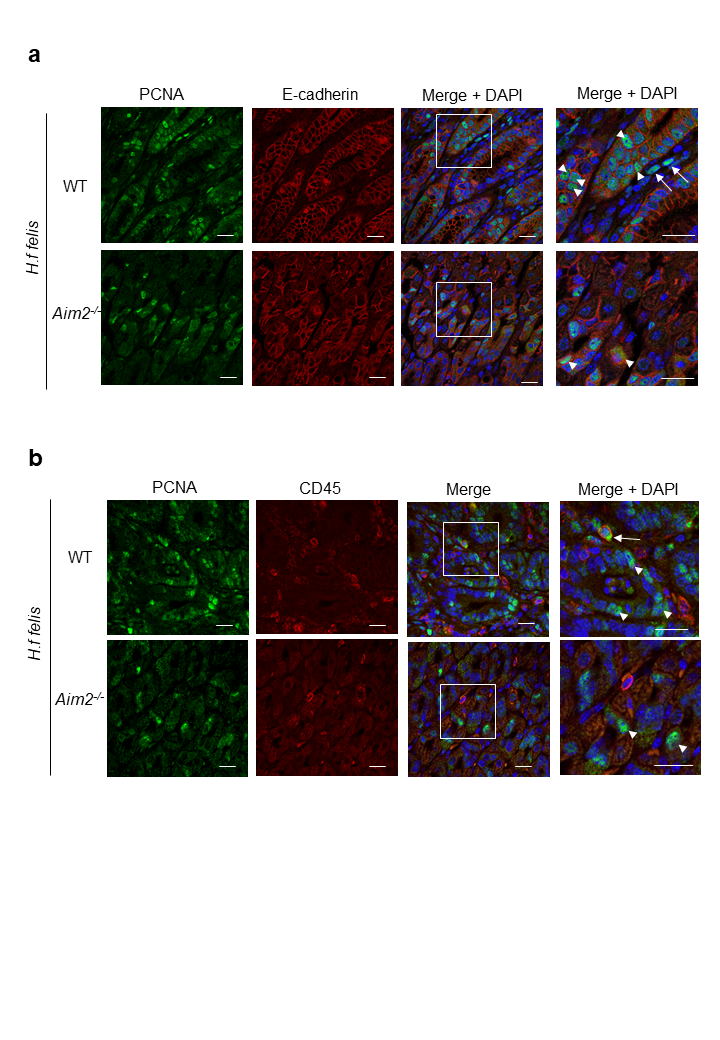
**Supplementary figure 3**: **Co-localisation of proliferating PCNA-positive epithelial and immune cells in *H. felis*-infected mouse gastric tissues.** Multicolour immunofluorescence staining for PCNA (green) and E-cadherin (red) **(a)**, and PCNA (green) and CD45 (red) **(b)**, in *H. felis* infected WT and *Aim2*^-/-^ mouse gastric tissue cross-sections. In **(a)**, arrowheads indicate dual stained epithelial cells while arrows indicate E-cadherin negative, PCNA-only stained immune cells. In **(b)**, arrows indicate dual stained immune cells while arrowheads indicate CD45 negative, PCNA-only stained epithelial cells. Scale bars = 20 μm.


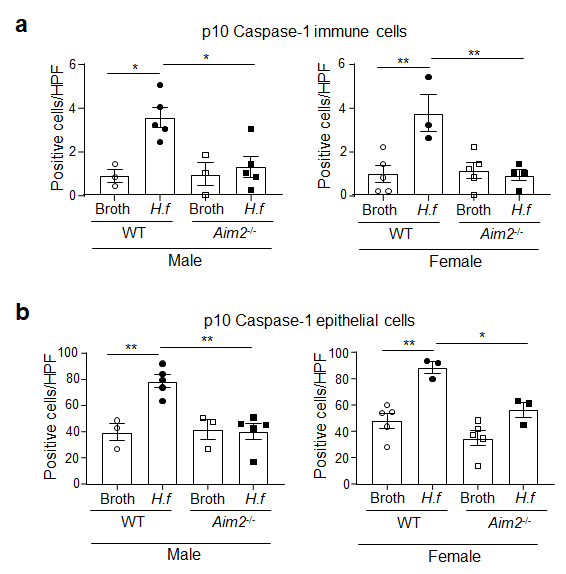


**Supplementary figure 4**: Reduced Caspase-1 activity in *Aim2^-/-^* *H. felis* mouse stomachs is independent of gender bias. (**a, b**) Graphs depict quantification of positively immunostained cleaved (p10) Caspase-1 **(a)** immune cells and **(b)** epithelial cells (from main Figure 6b) in gastric tissue sections from broth and *H. felis* gavaged WT and *Aim2*^-/-^ mice that have been segregated into males and females (n = 3–5/group). **P* < 0.05, ***P* < 0.01; One-way ANOVA with multiple comparisons.


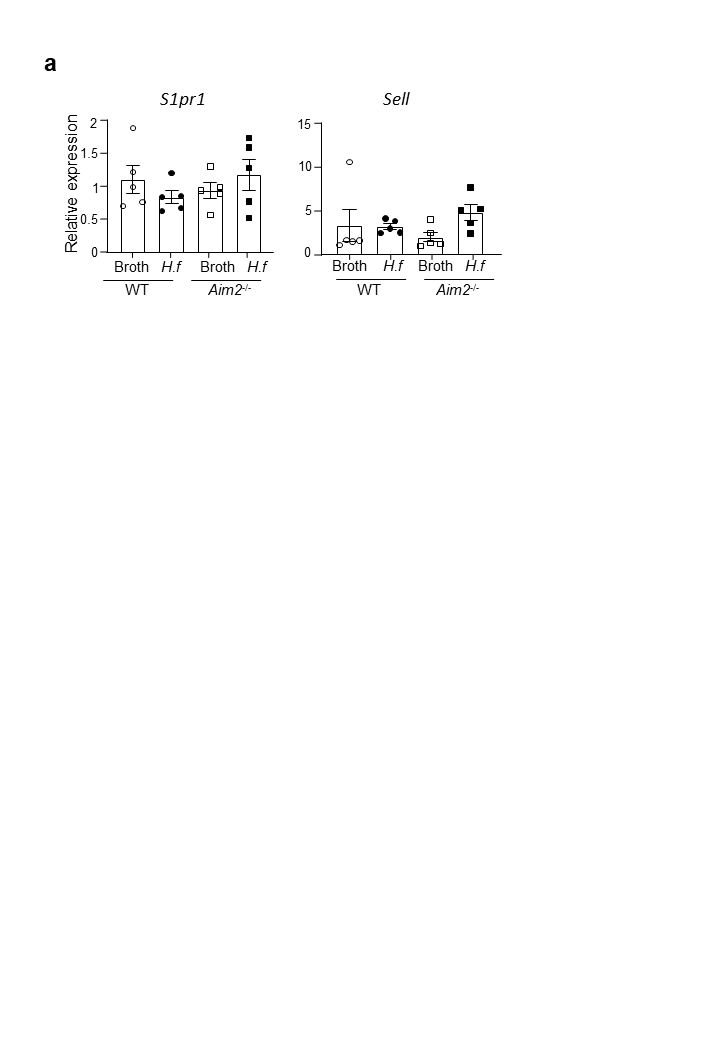


**Supplementary figure 5:** Gene expression analysis of selected immune homing receptors in *H. felis*-infected mouse gastric tissues. qPCR analysis for homing receptor genes in the indicated mouse gastric tissue samples (n = 5/group). Expression data are normalised to the *Rn18s* housekeeping gene.

**Supplementary table 1:** Gastritis patient biopsies with indicated *Helicobacter pylori* status and score of inflammation severity, N = normal, M = moderate, S = severe, Met = metaplasia. n.a = not available.

| **Patient ID** | ***Helicobacter* Status** | **Inflammation Status** | **Gender** | **Age** |
| --- | --- | --- | --- | --- |
| 6 | Negative | N | M | 46 |
| 26 | Negative | M/Met | F | 60 |
| 45 | Negative | N | F | 56 |
| 50 | Negative | N | n.a | n.a |
| 63 | Negative | N | M | 67 |
| 67 | Negative | M | n.a | n.a |
| 55 | Negative | M | n.a | n.a |
| 52 | Negative | N | n.a | n.a |
| 9 | Negative | M | M | 71 |
| 16 | Negative | M | F | 74 |
| 29 | Negative | S | n.a | n.a |
| 32 | Negative | M | F | 57 |
| 3 | Negative | N | M | 67 |
| 14 | Negative | S | F | 53 |
| 31 | Negative | M | M | 45 |
| 18 | Negative | S/Met | M | 73 |
| 28 | Negative | S | M | 42 |
| 3 | Negative | N | n.a | n.a |
| 21 | Positive | S/Met | F | 59 |
| 12 | Positive | S/Met | M | 64 |
| 39 | Positive | S/Met | F | 68 |
| 54 | Positive | S | M | 34 |
| 25 | Positive | S | n.a | n.a |
| 68 | Positive | M | F | 63 |
| 37 | Positive | M | M | 48 |
| 46 | Positive | M | M | 72 |
| 22 | Positive | S | M | 49 |
| 41 | Positive | S | M | 70 |
| 61 | Positive | S | F | 51 |
| 43 | Positive | S/Met | F | 74 |
| 24 | Positive | S | F | 72 |

**Supplementary table 2:** Primer sequences used for qPCR gene expression assays.

| **Species** | **Primer Name** | **Primer Sequence** |
| --- | --- | --- |
| **HUMAN** | *18S* Forward  *18S* Reverse  *PYCARD* Forward  *PYCARD* Reverse | 5’- CGGCTACCACATCCAAGGAA -3’  5’- GCTGGAATTACCGCGGCT -3’  5’- GCACTTTATAGACCAGCACCG -3’  5’- GGCTGGTGTGAAACTGAAGA -3’ |
|  | *CASP1* Forward  *CASP1* Reverse  *AIM2* Forward  *AIM2* Reverse  *NLRP1* Forward  *NLRP1* Reverse  *NLRP3* Forward  *NLRP3* Reverse  *NAIP* Forward  *NAIP* Reverse  *NLRP6* Forward  *NLRP6* Reverse  *NLRP12* Forward  *NLRP12* Reverse  *NLRC4* Forward  *NLRC4* Reverse | 5’- CTTCCTTTCCAGCTCCTCAGGCA -3’  5’- CGTGTGCGGCTTGACTTGTCC -3’  5’- CATCTGCAGCCATCAGAAAT -3  5’- CGCTTCTGAAACCCTTCTCT -3’  5’- CAGGCAGCACAGATCAACAT -3’  5’- GTGACCTTGAGGACGGAGAA -3’  5’- AAGGGCCATGGACTATTTCC -3’  5’- GACTCCACCCGATGACAGTT -3’  5’- ACTTGCGTCCTTCAGGAACTGG -3’  5’- TTCACATTTGGGGAACCATTTGG -3’  5’- GACGCTGCTCTCCGTGTC -3’  5’- GTGAAGCGCTTGGTGATCTT -3’  5’- CGGCACCAACCCACATCTGG -3’  5’- GGTCTGGAGCTTGCACGTGG -3’  5’- TAGCCGAGCCCTTATTCAAA -3’  5’- ACCTTCTCGCAGCAAATGAT -3’ |
| ***Mouse*** | *18S* Forward  *18S* Reverse  *Sell* Forward  *Sell* Reverse  *S1pr1* Forward  *S1pr1* Reverse    *Casp1* Forward  *Casp1* Reverse | 5’- GTAACCCGTTGAACCCCATT -3’  5’- CCATCCAATCGGTAGTAGCG -3’  5’- TGCAGAAACACAGTGTGGAGCA -3’  5’- AGAAATGCCAGCCCCGAGA -3’  5’- GCGCGGTGTAGACCCAGAGT -3’  5’- GAGGGCGAGGTTGAGTGAGC -3’  5’- ACGCCATGGCTGACAAGATCCTG -3’  5’- GGTCCCGTGCCTTGTCCATAGC -3’ |
|  | *Aim2* Forward  *Aim2* Reverse  *Nlrp1* Forward  *Nlrp1* Reverse  *Nlrp3* Forward  *Nlrp3* Reverse  *Naip1* Forward  *Naip1* Reverse  *Nlrp6* Forward  *Nlrp6* Reverse  *Nlrp12* Forward  *Nlrp12* Reverse  *Nlrc4* Forward  *Nlrc4* Reverse  *Pycard* Forward  *Pycard* Reverse  *Tnfa* Forward  *Tnfa* Reverse  *Ifng* Forward  *Ifng* Reverse  *Il11* Forward  *Il11* Reverse  *Cxcl16* Forward  *Cxcl16* Reverse  *Cxcl1* Forward  *Cxcl1* Reverse  *Cxcl2* Forward  *Cxcl2* Reverse | 5’- GCTGGGTGGCGTCAGGAAGTTT -3’  5’- TCCATCTCACAGTCCCAGGATCA -3’  5’- ATGTGGACCCAACCTTCAAA -3’  5’- GTACGTGCTCCTGGAAAGGT -3’  5’- GCACCAACCGGAGCCTCACT -3’  5’- CAGCGCCCCAACCACAGTCT -3’  5’- AGTGGTGGGCTTGGGTAGGAGT -3’  5’- CCCTTGCACAGGCTGGCTGA -3’  5’- GCAGACGAGCTGCCTACTTT -3’  5’- GCTCCTGGTAACAGCTCCTG -3’  5’- GCCCAGTCCAACATCACTTT -3’  5’- CCTCTTTGAGCCAGACGAAG -3’  5’- GACGCTTTGACTCACCACAA -3’  5’- CGTTCAATGCAAAGAGGTCA -3’  5’- AGCCAGAACAGGACACTTTGTG -3’  5’- GGCTGGTGGTCTCTGCACGAA -3’  5’- CAAATTCGAGTGACAAGCCTG -3’  5’- GAGATCCATGCCGTTGGC -3’  5’- ACAATGAACGCTACACACTGCAT -3’  5’- TGGCAGTAACAGCCAGAAACA -3’  5’- CTGCACAGATGAGAGACAAAT -3’  5’- GAAGCTGCAAAGATCCCAATG -3’  5’- CAAGACCAGTGGGTCCGTGA -3’  5’- TGCTCGTGTCCGAAGGTGTC -3’  5’- CCTTGACCCTGAAGCTCCCT -3’  5’- CGGGTGCCATCAGAGCAGTCT -3’  5’- AACATCCAGAGCTTGAGTGTGA -3’  5’- TTCAGGGTCAAGGCAAACTT -3’ |
